# Predictors of long‐term success after high‐density mapping‐guided substrate ablation procedures for ventricular tachycardia in patients with ischemic cardiomyopathy

**DOI:** 10.1002/joa3.13175

**Published:** 2024-11-01

**Authors:** J. C. Balt, B. G. S. Abeln, V. F. van Dijk, M. C. E. F. Wijffels, M. Liebregts, L. V. A. Boersma

**Affiliations:** ^1^ St. Antonius Hospital Nieuwegein The Netherlands; ^2^ Amsterdam University Medical Centers Amsterdam The Netherlands

**Keywords:** ablation, high‐density mapping, substrate mapping, ventricular tachycardia

## Abstract

**Introduction:**

High‐density (HD) substrate mapping may increase success of catheter ablation targeting ventricular tachycardia (VT). However, despite its use, recurrent VT is not uncommon. We aim to investigate factors that are associated with outcomes after HD mapping‐guided substrate ablation procedures for VT in patients with ischemic cardiomyopathy.

**Methods:**

Observational cohort study includes patients with ischemic heart disease who underwent HD mapping‐guided substrate ablation of VT. Baseline and procedural characteristics were associated with outcomes after VT ablation.

**Results:**

VT ablation employing HD mapping was performed in 80 patients. Median follow‐up was 2.3 years. VT‐free survival at one, two, and five years were 65%, 49%, and 40%. One‐, two‐, and five‐year implantable cardioverter defibrillator (ICD) shock‐free survival rates were 90%, 81%, and 70%. Complications occurred in 3 patients (3.8%, 1 vascular, 2 tamponades). Left ventricular ejection fraction (LVEF) and 45 W (vs. 35 W) ablation power were associated with VT‐free survival. High ablation power was also associated with shock‐free survival. All‐cause mortality during follow‐up was associated with higher age, the presence of chronic obstructive pulmonary disease (COPD), LVEF, and urgent ablation.

**Conclusions:**

In patients with ischemic cardiomyopathy that had HD substrate mapping‐guided VT ablation, ablation power was associated with both VT‐free and shock‐free survival, underlining the importance of effective target elimination. All‐cause mortality during follow‐up was associated with several factors (age, COPD, LVEF, and urgent vs. elective ablation), which could be used to guide patient selection for VT ablation.

## Graphical abstract

In 80 patients with ischemic cardiomyopathy that had high‐density substrate mapping‐guided ablation for ventricular tachycardia, two‐year VT‐free and ICD shock‐free survival was 49 and 81%, respectively. Higher left ventricular ejection fraction and higher ablation power are associated with VT‐free survival. All‐cause mortality during follow‐up is associated with higher age, the presence of chronic obstructive pulmonary disease (COPD), LVEF, and urgent ablation.

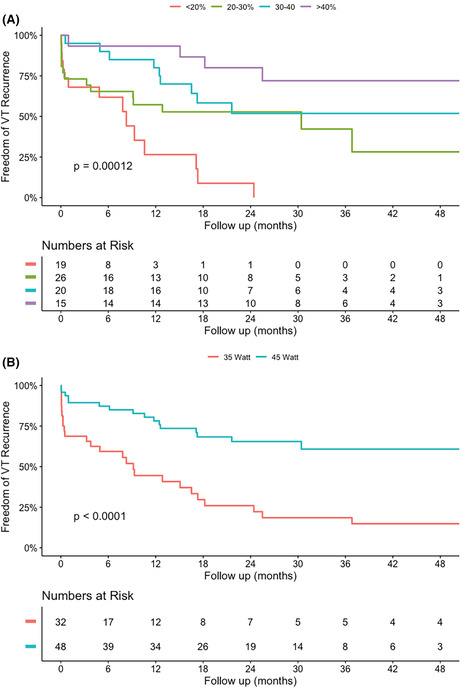

## 1 INTRODUCTION

Radiofrequency (RF) ablation for the treatment of ventricular tachycardia (VT) has been shown to reduce episodes of VT and shocks from implantable cardioverter defibrillators (ICDs) in ischemic cardiomyopathy.^1^, ^2^ However, VT recurrence after catheter ablation remains high.^3^, ^4^ Freedom from recurrent VT is thought to depend on successful mapping and ablation of areas of slow conduction or reentry circuits in the myocardial scar.^5^ High‐density (HD) substrate mapping to assess location and characteristics of the myocardial scar has demonstrated promising but varying results.^5^, ^6^, ^7^ The aim of the present study was to identify patient and procedural characteristics that determine the outcome of HD substrate mapping and ablation.

## 2 METHODS

Consecutive patients who underwent multi‐electrode substrate mapping during first‐time ablation for ischemic VT were included. Data on baseline characteristics, procedural characteristics, acute procedural success, safety, arrhythmic outcomes, and mortality at follow‐up were collected. Univariate and multivariable analyses were performed to determine the association between patient and procedural characteristics, and VT recurrence, ICD shocks, and survival. The institutional medical ethics committee approved the conduct of this study and issued a waiver for informed consent for retrospective analysis of our VT database.

### 2.1 Ablation procedures

The ablation procedures were performed under uninterrupted use of oral anti‐arrhythmic drugs. Intravenous procainamide was stopped 12 h prior to ablation. The procedures were performed under uninterrupted direct oral anticoagulants or continued oral anticoagulation with vitamin K‐antagonists (target international normalized ratio between 2.0 and 3.0). Intravenous heparin was used during the procedures to maintain a target‐activated clotting time of ≥300 s. Ablation procedures were performed under general anesthesia or conscious sedation with diazepam and fentanyl.

A Josephson quadripolar catheter was deployed into the apex of the right ventricle, and a linear quadripolar catheter was inserted into the coronary sinus. The left ventricle was accessed via a retrograde transaortic approach, using a transseptal puncture (Agilis™, Abbott Cardiovascular, Plymouth, MN, USA), or both. Ablation was performed using irrigated, contact force sensing RF ablation catheters (TactiCath™, Abbott Cardiovascular; or ThermoCool®, Biosense Webster, Irvine, CA, USA). Bipolar voltage mapping was performed with the following settings: normal tissue (>1.5 mV) and dense scar (<0.5 mV). Intracardiac signals were filtered at 30–400 Hz. Irrigated Ablation was performed with 35 or 45 W and 30°C for 30–60 s per application.

### 2.2 Substrate mapping

Low‐voltage areas were identified using electroanatomic maps. Maps were constructed using HD mapping systems (EnSite Precision or EnSite X, Abbott Cardiovascular; or CARTO 3, Biosense Webster) and multi‐electrode mapping catheters (Advisor™ HD Grid Mapping Catheter, Sensor Enabled, Abbott Cardiovascular; or PentaRay® NAV Catheter, Biosense Webster). In cases that were performed with the HD Grid catheter, the HD wave algorithm was used to automatically register the maximum voltage from two orthogonal electrodes. VT induction was not performed pre‐ablation. Fractionated, delayed, or abnormal electrograms, low‐amplitude, high‐frequency signals, occurring in or after the terminal QRS complex, crossing the baseline multiple times were identified, manually tagged on the electroanatomical map. In addition, automatic annotation of late potentials, defined as the latest deflection in the QRS window, was used to create late potential maps. During VT, diastolic potentials were also marked on the map. All marked anatomical locations (which invariably showed overlap) were subsequently ablated during sinus rhythm. The aim was to eliminate all abnormal potentials. Activation maps were constructed of spontaneously occurring (or induced, see below) and hemodynamically tolerated VTs. The critical isthmus of the VT circuit was identified. In addition, diastolic potentials were identified and manually annotated. On operator discretion, entrainment mapping was performed. The critical isthmus and diastolic potentials were subsequently ablated. In case of hemodynamically instable VT, overdrive pacing or cardioversion was performed and pacemapping and ablation was applied. Remaps were performed after ablation. Remaining late potentials were subsequently ablated.

After ablation, non‐inducibility was assessed using repeated programmed extra stimulation from the right ventricle apex, up to three extrastimuli or until refractoriness.

If pre‐procedural cardiac magnetic resonance images were available, they were used to generate a pre‐procedural three‐dimensional fibrosis map using ADAS 3D LV software (Galgo Medical, Barcelona, Spain). The pre‐procedural magnetic resonance imaging‐based fibrosis maps were merged with the procedural electroanatomic maps.

### 2.3 Follow‐up

All patients were invited for outpatient clinic visits (including 12‐lead ECG assessment) at 3 and 12 months post‐ablation, and at 12‐month intervals thereafter. Interrogation of ICDs was performed at 6‐month intervals to assess the occurrence of any VTs or anti‐arrhythmic therapies (including anti‐tachycardia pacing (ATP) and ICD shocks). Continuation of anti‐arrhythmic drugs was at the discretion of the treating cardiologist.

### 2.4 Outcomes

Endpoints were procedural success defined as non‐inducibility of any VT, acute procedural complications, adverse events within 30 days, recurrent VT (documented VT > 30s or terminated by ATP or ICD shock) and appropriate ICD shock during follow‐up, all‐cause mortality, cardiac (non‐arrhythmic) death, and arrhythmic death.

### 2.5 Data collection

Data were collected using the Research Electronic Data Capture (REDCap) system. Custom‐built electronic case report forms were designed to capture the data for this registry, incorporating real‐time data validation, integrity checks, and to facilitate audits to assure data quality.

### 2.6 Statistical analysis

Data are presented as mean and standard deviation for normally distributed continuous variables, median, and interquartile range for not normally distributed variables and as numbers and percentages for categorical variables. As this was an exploratory observational study, no formal hypothesis, statistical plan, or power calculation were performed. Event‐free survival was graphically depicted using the Kaplan–Meier method. Univariate and multivariable Cox‐regression models were fitted to identify predictors of recurrent events during follow‐up. Variables for inclusion in the multivariable model were selected based on their *p* value ≤0.1 in the univariate model. All tests were two‐tailed, and the limit for statistical significance was set at *p* < 0.05. Statistical analysis was performed using R (R Foundation for Statistical Computing, Vienna, Austria).

## 3 RESULTS

Between January 2016 and June 2022, first time VT ablation using a HD mapping system was performed in 80 patients. Baseline parameters are shown in Table 1. Procedural characteristics are shown in Table 2.

**TABLE 1:** Baseline characteristics.

	*n* = 80
Age, years	69 ± 10
Male sex	73 (91.3)
BMI, kg/m^2^	27 ± 4
Hypertension	37 (46.2)
Diabetes mellitus	11 (13.8)
Renal dysfunction	36 (45.0)
COPD	18 (22.5)
Atrial fibrillation	27 (33.8)
LVEF, %	31 ± 10
Infarct location	
Anterior	26 (32.5)
Posterior	32 (40.0)
Inferoseptal	22 (27.5)
Clinical setting	
Elective	42 (52.5)
Urgent	38 (47.5)
Anti arrhythmic drugs	
Class I	1 (1.2)
Class II	11 (13.8)
Sotalol	23 (28.7)
Amiodarone	45 (56.2)

**TABLE 2:** Procedural characteristics.

	*n* = 80
Approach	
Retrograde	1 (1.2)
Transseptal	73 (91.2)
Both	6 (7.5)
MRI integration	21 (26)
Mapping catheter	
PentaRay	30 (38)
HD Grid	50 (62)
Points per map	14,529 ± 7811
Ablation power	
35 Watt	32 (40.0)
45 Watt	48 (60.0)
VT induced	59 (74)
Number of induced VTs	2 [1–4]
Inducibility after ablation	
VT still inducible	24 (30)
VT non‐inducible	38 (48)
Not assessed	18 (23)
Procedure time, minutes	159 ± 27
Ablation time, minutes	28 ± 10
Fluoroscopy time, minutes	26 ± 8
Dose Area Product, Gy·cm^2^	50 ± 23
Complications	
Vascular	1 (1)
Tamponade	2 (3)
Cerebrovascular accident	0
Procedural death	0

Procedural complications occurred in 3 patients (3.8%, 1 vascular, 2 tamponades). No strokes and no procedural death occurred.

Median follow‐up was 2.3 years (interquartile range 1.3–3.9 years). During follow‐up, 46% of patients used amiodarone (with or without beta‐blocker), 25% of patients used sotalol, and 29% used beta‐blockers only. In patients with VT recurrence during follow‐up, 51% of patients used amiodarone (with or without beta‐blocker), 24% of patients used sotalol, and 24% used beta‐blockers only. In comparison, patients without recurrence of VT, 41% used amiodarone (with or without beta‐blocker), 26% of patients used sotalol, and 33% used beta‐blockers only (not significant (NS)).

**FIGURE 1:**
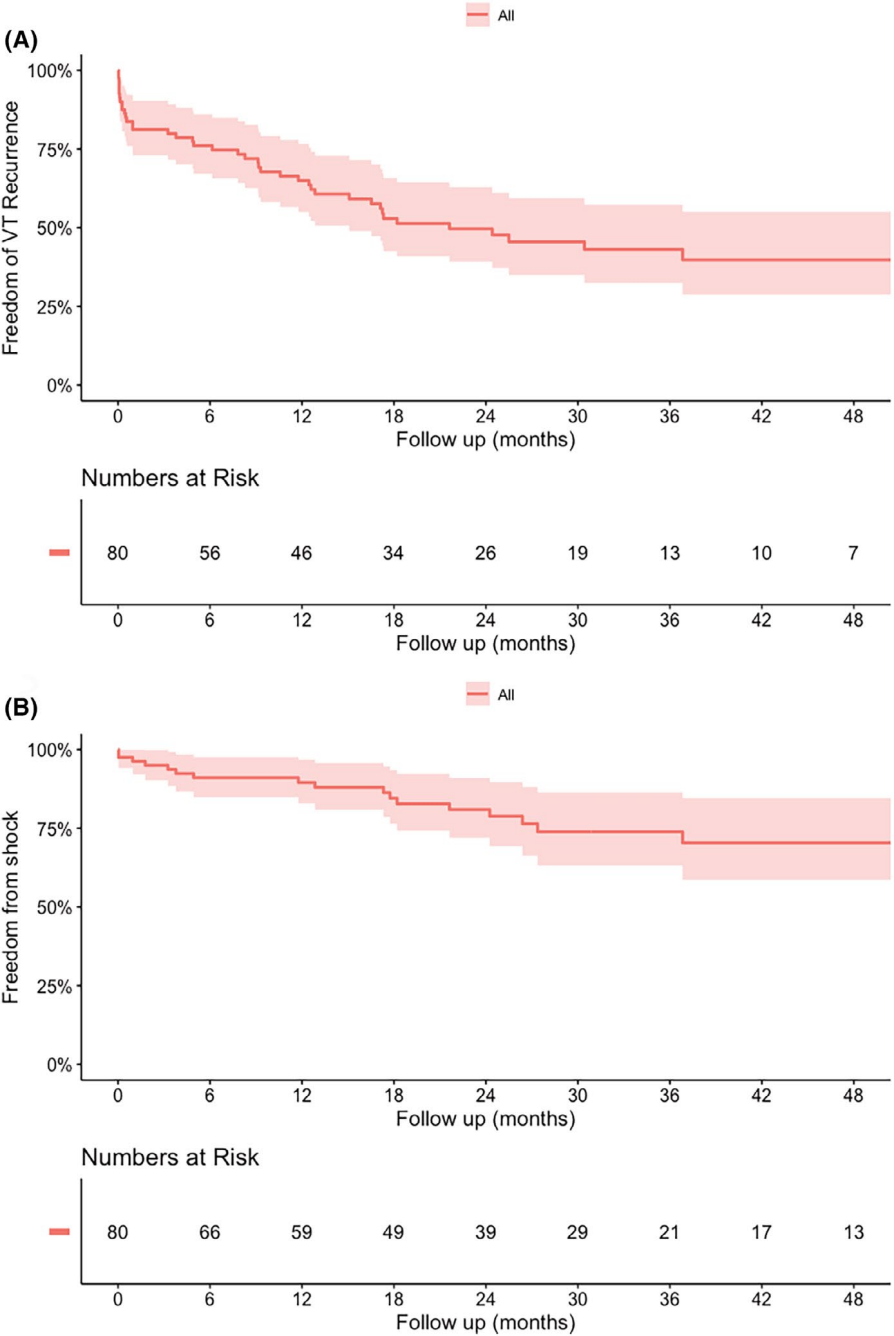
Arrhythmia outcomes. (A) Kaplan–Meier curve for VT recurrence. (B) Kaplan–Meier curve for ICD shock.

Kaplan–Meier curves for VT‐ and shock‐free survival during follow‐up are shown in Figure 1A,B. One‐, two‐, and five‐year VT‐free survival was 65%, 49%, and 40%. One‐, two‐, and five‐year shock‐free survival were 90%, 81%, and 70%.

**FIGURE 2:**
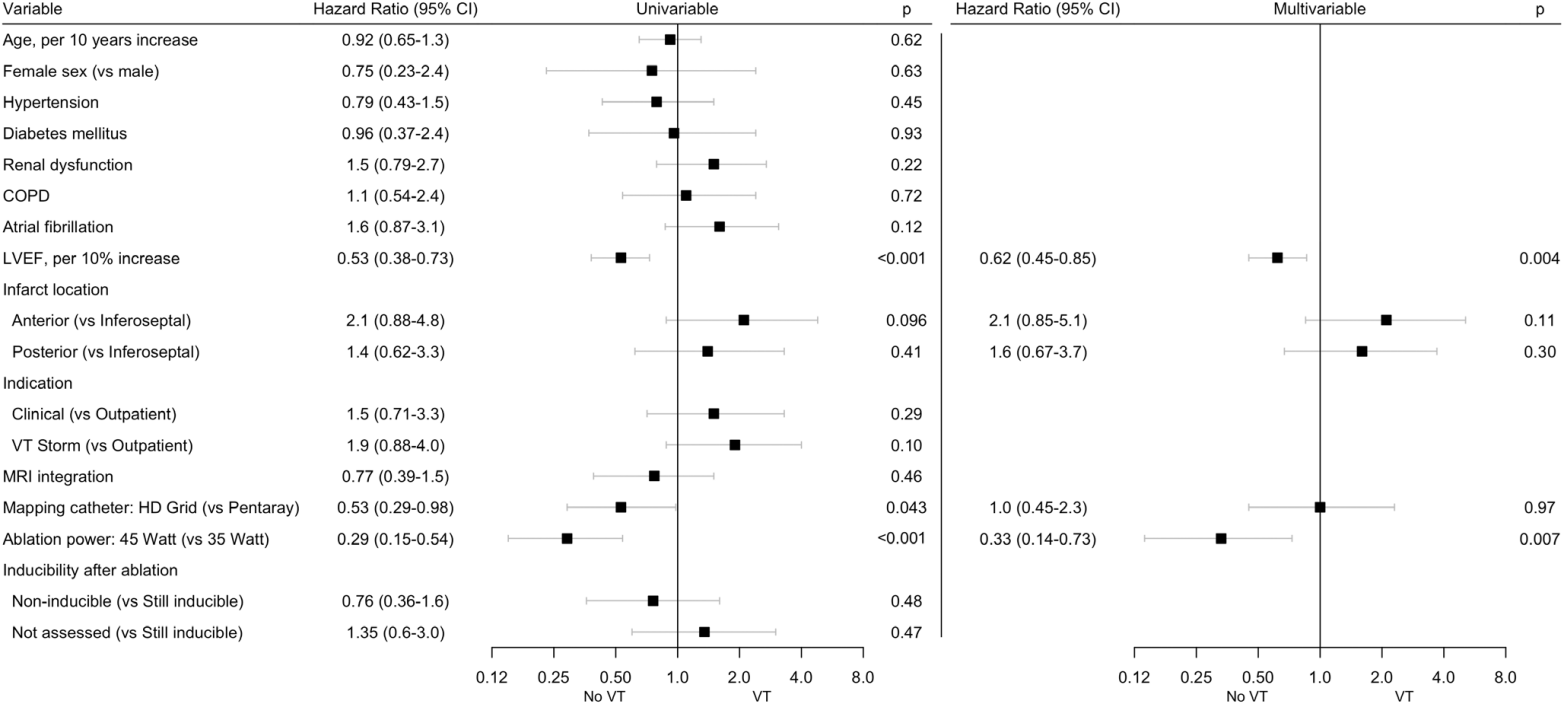
Cox proportional hazards regressions for VT recurrence.

**FIGURE 3:**
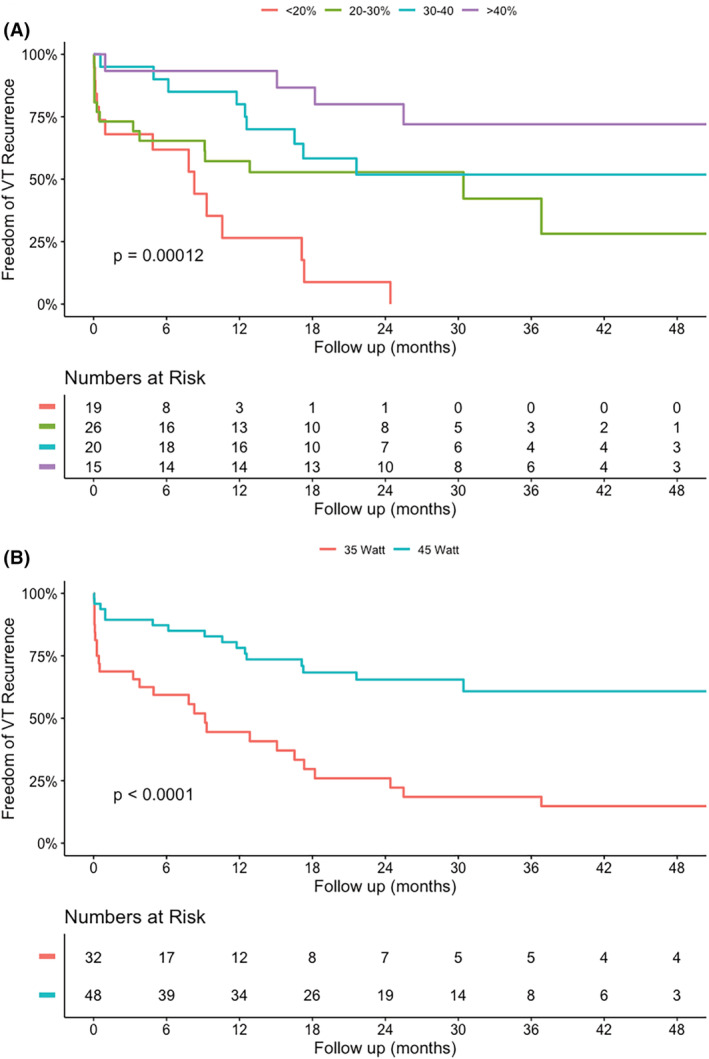
Kaplan–Meier curves for VT recurrence, stratified for LVEF (A) and ablation power (B).

Univariate and multivariable associations with recurrent VT are shown in Figure 2. In multivariable analysis, a higher left ventricular ejection fraction (LVEF), and the use of 45 W ablation power (compared to 35 W) were significantly associated with VT‐free survival. Kaplan–Meier curves of VT‐free survival, stratified for LVEF and ablation power used, are shown in Figure 3. Of the 28 patients with VT recurrence at 1 year, a reduction of more than 50% of the number of VTs was achieved in 13 patients (46% of patients with VT recurrence).

**FIGURE 4:**
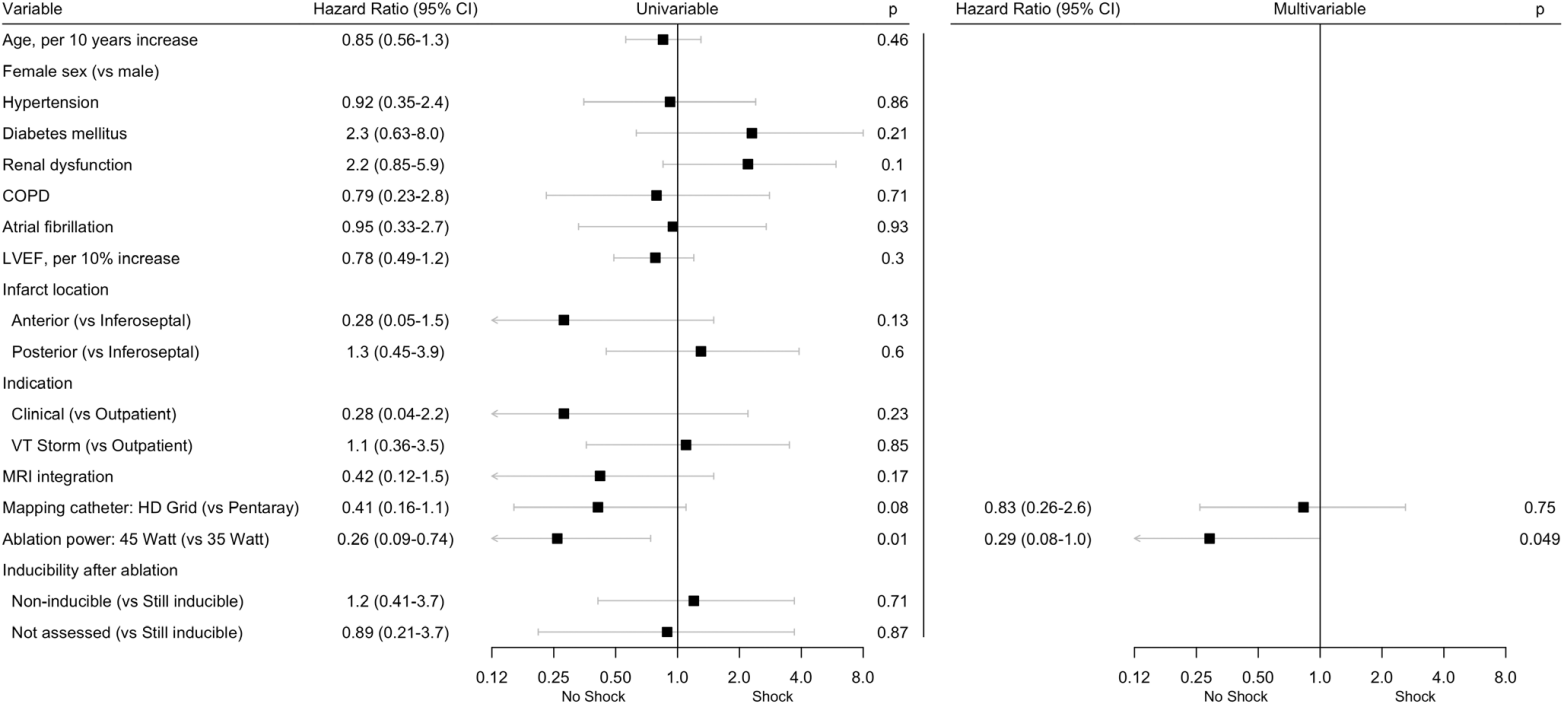
Cox proportional hazards regression for ICD shock during follow‐up.

Univariate and multivariable associations with recurrent shock are shown in Figure 4. Ablation power of 45 W (compared to 35 W) was significantly associated with freedom from ICD shocks in multivariable analysis. Kaplan–Meier curves of shock‐free survival, stratified for LVEF and ablation power used, are shown in Figure S1.

A total of 22 patients (28%) underwent one or more repeat VT ablation(s). In 5 repeat VT ablations, epicardial ablation was performed. After repeat ablation, 15 (68%) and 18 (82%) patients remained free from recurrent VT and shock, respectively.

One‐, two‐, and five‐year freedom from all‐cause mortality was, 81, 77, and 62%, respectively.

One‐, two‐, and five‐year freedom from cardiac (non‐arrhythmic) mortality was 87, 84, and 77%, respectively. One‐, two‐, and five‐year freedom from arrhythmic death was 96, 93, and 93%, respectively.

**FIGURE 5:**
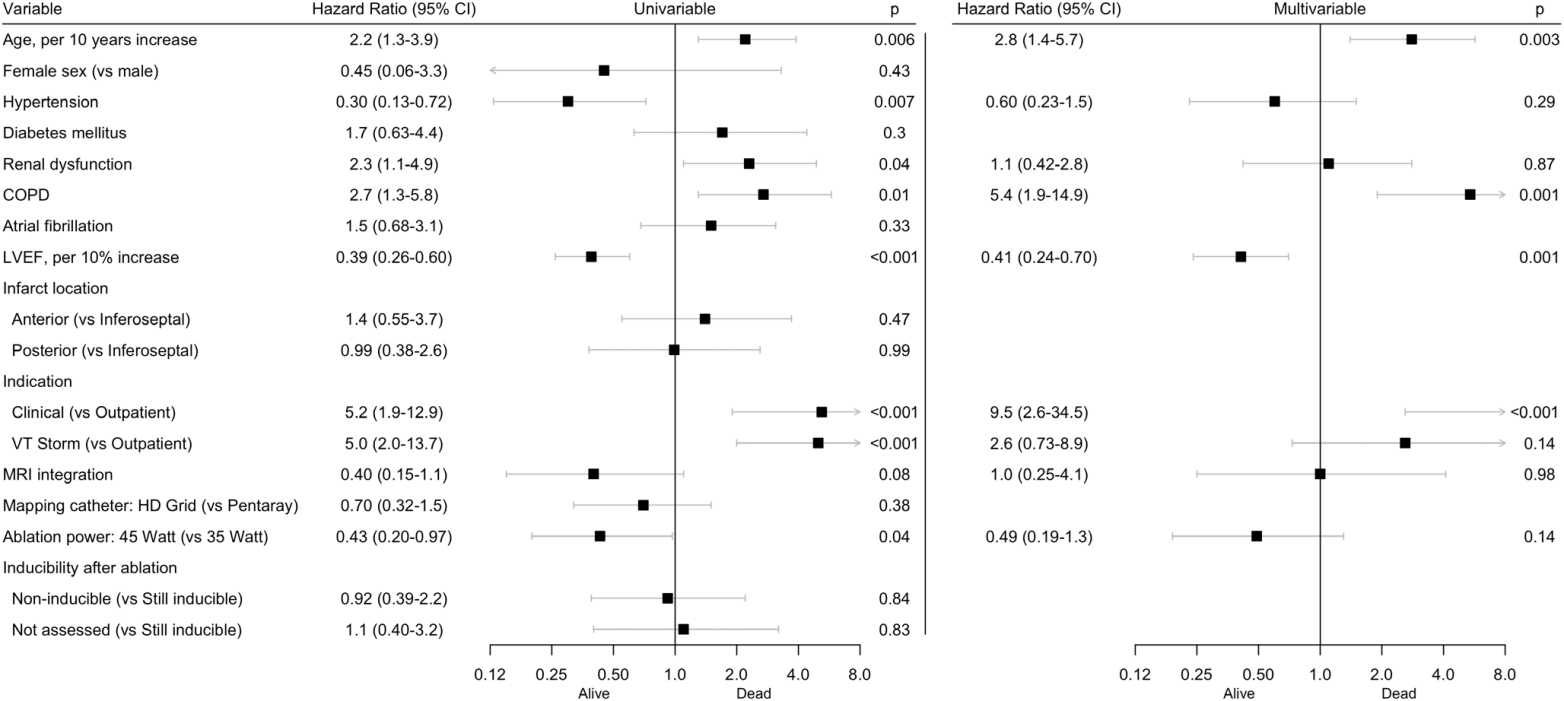
Cox proportional hazards regression for all‐cause mortality.

Univariate and multivariable associations with all‐cause mortality are shown in Figure 5. In multivariable analysis, higher age, the presence of chronic obstructive pulmonary disease (COPD), lower LVEF, and urgent VT ablation (compared to elective) were associated with all‐cause mortality. Kaplan–Meier curves of all‐cause mortality, stratified for LVEF and setting of VT ablation, are shown in Figure S2.

**FIGURE 6:**
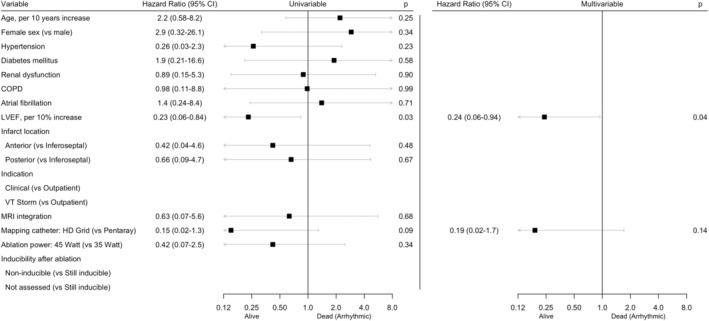
Cox proportional hazards regression for arrhythmic mortality.

Univariate and multivariable associations with arrhythmic mortality are shown in Figure 6. In multivariable analysis, only lower LVEF was significantly associated with arrhythmic mortality.

## 4 DISCUSSION

HD substrate mapping to assess the location and characteristics of the myocardial scar in patients with ischemic VT has demonstrated promising but varying results.^8^, ^9^, ^10^ The aim of the present study was to identify patient and procedural characteristics that determine the outcome of HD substrate mapping and ablation.

The main finding of the present study is that after HD substrate mapping, high (45 W) ablation power was associated with VT‐free and shock‐free survival. A higher LVEF was also associated with VT‐free survival. Higher age, the presence of COPD, lower LVEF, and urgent ablation were independently associated with all‐cause mortality.

The study population consisted of consecutive patients with ischemic cardiomyopathy undergoing a first VT ablation and resembles patients in most VT studies, being mostly male, with a low ejection fraction and a high‐risk profile. Half of the procedures were performed in an urgent setting, for example, patients with repeated ICD shocks or VT storm. Also, half of the patients was on amiodarone at baseline (Table 1). Ablations were mostly performed by transseptal approach. Complications were comparatively infrequent,^4^ and no permanent sequelae occurred (Table 2).

### 4.1 Effective substrate mapping‐guided ablation

VT in ischemic cardiomyopathy is typically caused by reentry. The main strategy of VT ablation is to identify and eliminate critical parts of the reentry circuit. HD mapping has emerged as an alternative to a classic VT mapping approach, which employs entrainment mapping, pace mapping, and single tip activation mapping. HD mapping systems can be used for substrate mapping to identify areas of slow conduction. These areas are characterized by low amplitude, abnormal, fragmented potentials that occur late in the QRS complex during sinus rhythm or ventricular pacing. The locations of the abnormal electrograms are marked and subsequently ablated.^11^ Substrate mapping can be advantageous in patients with fast VTs that are not hemodynamically tolerated.

When mapping low‐voltage, abnormal, and fragmented electrograms, it is beneficial to use small electrodes.^12^ Catheters equipped with multiple small electrodes can overcome the inherent disadvantages of conventional mapping, cover larger areas in a shorter timeframe, and can also assist in mapping activation fronts.^13^, ^14^ Indeed, multi‐electrode mapping demonstrated better discrimination of local abnormal electrograms and conducting channels.^15^ The effectiveness of HD Grid mapping of both late potentials and diastolic potentials during VT was demonstrated by Okubo et al.^16^

An early multi‐electrode substrate mapping strategy study showed one‐ and two‐year VT recurrence of 40% and 46%, respectively.^12^ A favorable effect of HD Grid substrate mapping was shown in a study by Proietti et al.^17^ Shock‐free survival was 100% after a mean follow‐up of 1 year, and this was significantly higher compared to patients in which PentaRay, duodeca, or single tip mapping were performed. In fact, a correlation was found between a favorable outcome and mapping density. In contrast, Maagh et al. found no such a difference.^8^ These authors compared single tip mapping with the Navistar vs. multi‐electrode mapping with the PentaRay (75% of patients had ischemic cardiomyopathy). Clinical VT ablation and ablation of late potentials were applied in both groups. After 12‐month follow‐up, VT‐free survival was 79%, with no difference between the two groups.^8^ In a previous study, we did not observe better VT‐free survival when HD mapping was applied compared to single tip mapping and ablation.^9^ Several factors such as inaccurate mapping despite HD mapping tools and substrate mapping during sinus rhythm instead of HD activation mapping may have played a role. Alternatively, it is conceivable that accurate mapping, but inadequate ablation may have been the cause of not capitalizing on HD mapping tools in the setting of VT ablation.

### 4.2 Adequate VT target elimination

The inability to reach ablation targets that lie deep within the (left) ventricular wall has long been recognized as a cause for VT recurrence after ablation.^18^ Persistence of late potentials after ablation and inability to achieve non‐inducibility may reflect inadequate elimination of isthmuses and areas of slow conduction.^19^, ^20^

Several strategies have been attempted to deliver effective ablation lesions deep into the ventricular substrate, such as needle ablation, bipolar ablation, half saline irrigation, higher power settings, (ultra‐low temperature) cryoablation, and even stereotactic radio‐ablation.^5^, ^21^, ^22^, ^23^

For RF ablation, factors that influence lesion size include power, contact force, and the duration of the application.^24^, ^25^, ^26^ Importantly, RF lesions deployed by similar duration, power, and contact force are smaller in low‐voltage areas compared to normal voltage areas.^27^ Caution is needed using higher power settings, increased contact force, or longer duration applications, because these may potentially cause unwanted side effects and complications, such as coagulum formation and steam pops. Also, half saline irrigation is associated with a 12% rate of steam pops, making this a powerful tool that needs to be handled with care.^28^

In the initial phase of irrigated RF ablation for VT, power settings described were around 35‐40 W.^29^, ^30^ In our center, ablation procedures between 2015 and 2018 were also performed with power set at 35 W, with 17 mL/min irrigation, for 30–60s per lesion. In later reports, power setting up to 50 W were described for irrigated RF.^16^, ^31^ No detrimental sequelae were reported. From October 2018 onward, we used 45 W with 30 mL/min irrigation, for 30–60s per lesion for VT ablation in the left ventricle, also in (the border zone of) infarcted myocardial tissue.

### 4.3 Predictors of outcome of substrate‐guided VT ablation

In our study, we found a higher LVEF and the use of 45 W ablation power to be independently and significantly associated with VT‐free survival. Shock‐free survival is independently associated with 45 W (vs. 35 W). This confirms the results of previous studies that reported lower LVEF (reflecting a more extensive arrhythmic substrate) and incomplete substrate ablation as predictors of VT recurrence.^20^, ^32^ Importantly, when we compared the number of RF applications (40 ± 13 vs. 42 ± 11, NS), ablation duration (26 ± 9 vs. 28 ± 11 min, NS) and contact force (12.2 ± 2.3 vs. 11.6 ± 2.9 g, NS) were similar in the 35 W and 45 W groups. From this analysis, we conclude that the RF energy delivered, not a difference in RF duration, or contact force may have resulted in more effective ablation.

A better outcome regarding VT‐free survival in the patients in which a higher power setting was used, confirms the abovementioned notion that after adequate identification of ablation targets, insufficient target elimination may be one of the causes of recurrent VT. No previous studies have reported on the effects of ablation power on VT recurrence and event‐free survival. The present study clearly points toward the need for effective ablation, after the substrate has been mapped in detail. Nevertheless, in our study, non‐inducibility was not different between the 35 W and 45 W group. A 62% non‐inducibility rate was reached in both groups. However, even though non‐inducibility has been shown to be associated with VT‐free survival,^19^ the predictive value of non‐inducibility had been shown to limited.^33^

Improved mapping may lead to better outcome,^17^, ^34^, ^35^ but tackling the third dimension (deep substrate ablation) remains challenging. Improved catheter design and even different ablation modalities warrant further exploration.^23^, ^36^

### 4.4 Patient selection and treatment aspects

In the present analysis, we found age, COPD, LVEF, and setting of the ablation (urgent versus elective) to be independently and significantly associated with all‐cause mortality. This confirms previous studies.^4^, ^33^ Factoring in these patient characteristics may help in identifying patients who may not benefit from VT ablation. In the present study, the PAINESD score, proposed in the study by Santangeli et al., was 11.3 ± 3.6 and was predictive of all‐cause death (HR 1.3 [1.2–1.5], *p* < 0.001) but not of VT recurrence (HR 1.1 [0.98–1.2], *p* = 0.13).

Two‐year freedom from all‐cause mortality was 77%, while freedom from arrhythmic death was 93%. Survival after VT ablation is clearly determined by non‐cardiac and cardiac non‐arrhythmic and less by arrhythmic death. This needs to be considered when planning ablation for ischemic VT, since these procedures are resource intensive.

We found no difference in the use of anti‐arrhythmic drug use between patients with and without recurrent VT; for instance, amiodarone (with or without beta‐blocker) was used in 51% and 41% of patients with and without recurrent VT (NS). However, in this retrospective analysis, relationships between AADs and events are difficult to interpret. For instance, recurrent VT during follow‐up may have caused continuation of amiodarone and sotalol therapy. On the other hand, a number of patients may have wished to continue amiodarone or sotalol, even in the absence of ICD shocks or VT recurrence.

### 4.5 Limitations

As this is a relatively small, retrospective study, in which randomization was not performed, the results have to be interpreted with caution and can only be hypothesis generating.

Our VT database focuses on clinical characteristics and follow‐up, and no detailed analysis of VT electrogram characteristics was performed.

## 5 CONCLUSIONS

In patients with ischemic cardiomyopathy, after HD substrate mapping and subsequent ablation, high (45 W) ablation power is associated with VT‐free and shock‐free survival, underlying the importance of effective target elimination. A higher LVEF is associated with VT‐free survival and freedom from arrhythmic death. Several factors (age, COPD, LVEF, and clinical setting) are independently associated with all‐cause mortality. These findings may impact patient selection for VT ablation.

## FUNDING INFORMATION

This research did not receive any specific grant from funding agencies in the public, commercial, or not‐for‐profit sectors.

## CONFLICT OF INTEREST STATEMENT

The authors of this manuscript declare that they have no conflicts of interest regarding the publication of this paper.

## ETHICS STATEMENT

The study was conducted in accordance with the Declaration of Helsinki, and the protocol was approved by the Ethics Committee and local institutional review board. For this retrospective analysis, no written patient approval was required by the Ethics Committee.

## Supporting information


**Data S1:** supporting Information.

**Data S2:** supporting Information.

## Data Availability

The data that support the findings of this study are available upon reasonable request.
