# Supplementary figures and images for: Predictors of long‐term success after high‐density mapping‐guided substrate ablation procedures for ventricular tachycardia in patients with ischemic cardiomyopathy
Source: J Arrhythm. 2024 Nov 1;40(6):1442–51. doi: 10.1002/joa3.13175 (PMC11632252; doi:10.1002/joa3.13175)

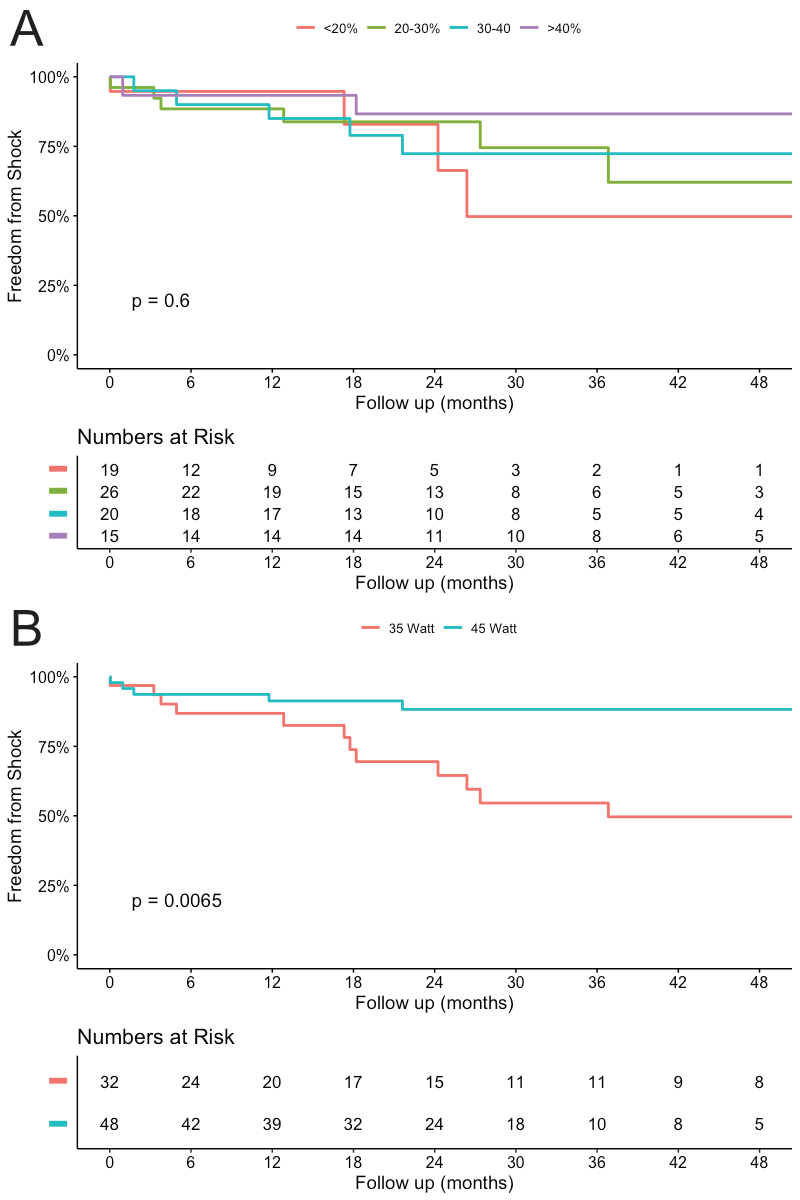

Supplement: Supplementary file 1 — Data S1: supporting Information. [file JOA3-40-1442-s001.tiff]

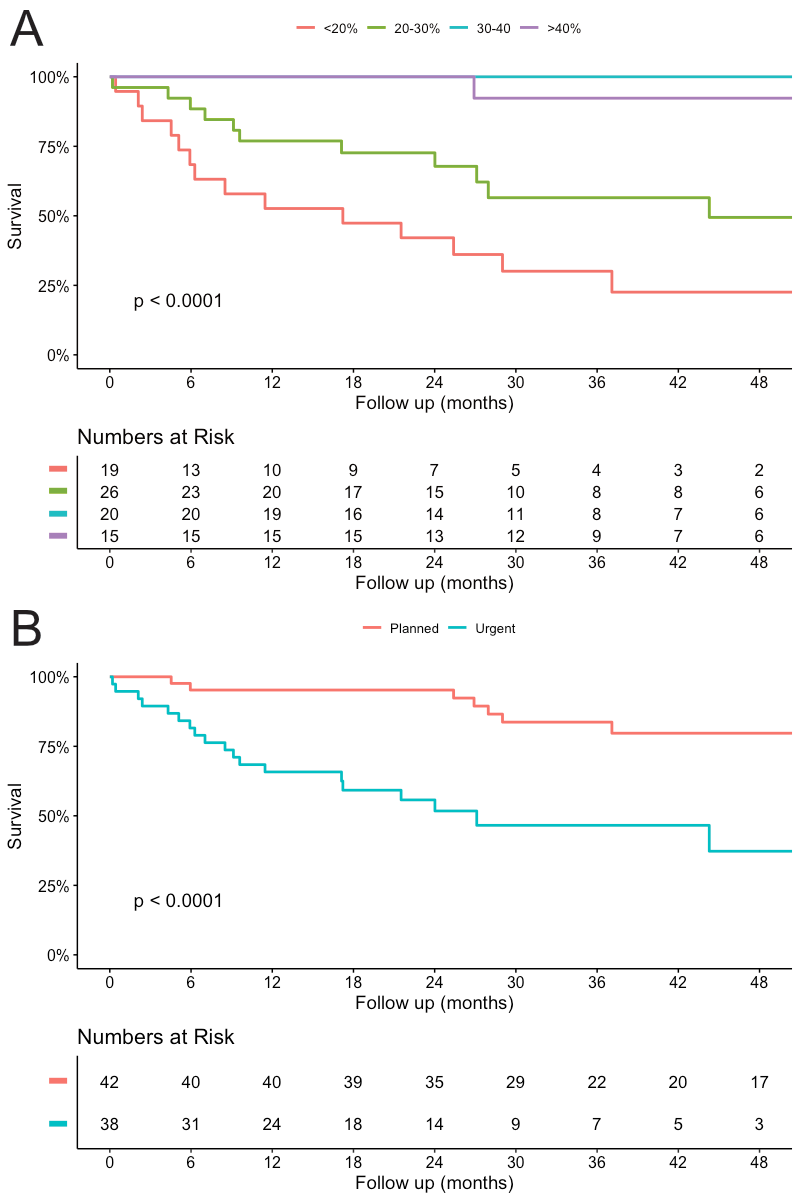

Supplement: Supplementary file 2 — Data S2: supporting Information. [file JOA3-40-1442-s002.tiff]
